# Plant-associated halotolerant bacteria improving growth of *Vicia faba* L. Mariout-2 under salinity conditions

**DOI:** 10.1038/s41598-024-66504-0

**Published:** 2024-07-20

**Authors:** Dalia Wael, Yasser El-Amier, Wesameldin I. A. Saber, Ashraf Elsayed

**Affiliations:** 1 Botany Department, Faculty of Science, Mansoura University, Mansoura, 35516 Egypt; 2 Microbial Activity Unit, Microbiology Department, Soils, Water and Environment Research Institute, Agricultural Research Center, Giza, 12619 Egypt

**Keywords:** Plant-associated halotolerant, Salinity stress, Plant growth promoting bacteria, *Vicia faba*, Seed priming, Biotechnology, Microbiology, Plant sciences, Environmental sciences

## Abstract

In this comprehensive investigation, we successfully isolated and characterized 40 distinct plant-associated halotolerant bacteria strains obtained from three halophytic plant species: *Tamarix nilotica*, *Suaeda pruinosa,* and *Arthrocnemum macrostachyum*. From this diverse pool of isolates, we meticulously selected five exceptional plant-associated halotolerant bacteria strains through a judiciously designed seed biopriming experiment and then identified molecularly. *Bacillus amyloliquefaciens* DW6 was isolated from *A. macrostachyum.* Three bacteria (*Providencia rettgeri* DW3*, Bacillus licheniformis* DW4*,* and *Salinicoccus sesuvii* DW5) were isolated for the first time from *T. nilotica, S. pruinosa* and *S. pruinosa*, respectively. *Paenalcaligenes suwonensis* DW7 was isolated for the first time from *A. macrostachyum*. These plant-associated halotolerant bacteria exhibited growth-promoting activities, including phosphate solubilization, nitrogen fixation, and production of bioactive compounds, i.e., ammonia, phytohormones, hydrogen cyanide, siderophores, and exopolysaccharides. A controlled laboratory experiment was conducted to reduce the detrimental impact of soil salinity. *Vicia faba* seedlings were inoculated individually or in mixtures by the five most effective plant-associated halotolerant bacteria to reduce the impact of salt stress and improve growth parameters. The growth parameters were significantly reduced due to the salinity stress in the control samples, compared to the experimental ones. The unprecedented novelty of our findings is underscored by the demonstrable efficacy of co-inoculation with these five distinct bacterial types as a pioneering bio-approach for countering the deleterious effects of soil salinity on plant growth. This study thus presents a remarkable contribution to the field of plant science and offers a promising avenue for sustainable agriculture in saline environments.

## Introduction

Approximately, 5.2 billion hectares (around 50%) of fertile land are affected by salinity^1^. Under salt stress, irrigation is a major problem of sustainable agriculture and consequently food production due to gradual salinization. The susceptibility of faba bean (*Vicia faba*) to salinity restricts or even prevents its cultivation in salinity-affected soil. There are several traditional techniques to overcome salinity problems in soil like drainage, leaching, reducing evaporation, applying chemical treatments, and a combination of these methods but all of these methods are time-consuming, high cost, unsustainable, and not ecofriendly. On the other hand, plant growth-promoting bacteria (PGPB) isolated from halophytes can provide sustainable mitigation of salinity stress of salinity-affected soil while they are cheap and eco-friendly sources.

Salt stress has threefold effects: it reduces water potentiality and causes ion imbalance or disturbance in ion homeostasis and toxicity. Chloride ions are toxic to plants and as the concentration of these ions increases, the plant is poisoned and dies. Salinity affects the production of crops especially faba beans by interfering with nitrogen uptake, reducing growth, and stopping plant reproduction^2^. Salinity stress has been studied in relation to regulatory mechanisms of osmotic and ionic homeostasis^3^. The response of plants to salinity stress may vary with the genotype; nevertheless, some general reactions occur in all genotypes. Salinity can affect plant physiological processes resulting in reduced growth and yield^4^. Increased tolerance to salinity stress in crop plants is necessary in order to increase productivity with limited water supplies and high salinity.

*Vicia faba* is regarded as one of Egypt's most strategic leguminous crops. Also, it’s important for soil fertility, human nutrition, animal feeding, and industrial purposes as functional food ingredients (potential foaming, emulsifying, and gelling agents that can be used for producing dairy and meat alternatives)^5^ and as a good source of vegetarian protein^6^. About 125,000 feddans of *Vicia faba* beans in Egypt were planted in 2020/2021 and this area achieved a sufficiency of 40%, the total production reached about 190 thousand tons, while the total consumption reached about 450 thousand tons annually, so the total production of the local market meets only about 40% of local consumption, so about 60% of the needs are imported from abroad^7^. Increasing faba bean production and improving yield quality are the major targets to meet the demand of the increasing Egyptian population^8^.

Plant-associated bacteria that associate with plants are of agricultural or environmental importance^9^. They are thought to be an excellent source of secondary metabolites, natural antibacterial agents, and compounds helping in salt tolerance^10–12^ through producing various types of phytohormones like gibberellin (GA), indole acetic acid (IAA), abscisic acid (ABA), cytokinins (CK), cofactor pyrroquinoline quinone (PQQ) and ethylene that promote plant growth^13^. Also, they produce secondary compounds such as exopolysaccharides^14^ and osmolytes (proline, trehalose, and glycine betaines)^13,15,16^, regulate plant defense systems, and activate plant’s antioxidative enzymes under salt stress^13^. In comparison to non-symbiotic plants, plant-associated and endophytic bacteria function as biological triggers that accelerate and intensify the stress response.

Plant-associated and endophytic bacteria with very low biomass compared to plants have the power to change the structure of plant communities, and this process would have continued throughout its expanding range^17^. For example, *Bacillus cereus* was isolated from leaves of *Stevia rebaudiana* and used in plant growth promotion and biocontrol^18^, *Enterobacter cloace*, *Klebsiella michiganensis* , and *Bacillus subtillus* were isolated from *Triticum vulgare* and wild *Phragmites australis* and used to enhance the growth and yield of maize^19^ and *Bacillus fexus* and *Brevibacillus parabrevis* were isolated from *Lotus glaber* and *Lotus creticus* and used to stimulate the growth and pigmentation of *Lactuca sativa* L^20^. Plant-associated halotolerant bacteria such as *Vibrio parahaemolyticus, Vibrio* sp and *V. alginolyticus* isolated from halophytes *Tamarix nilotica*^21^, *Bacillus subtilis* and *Bacillus pumilus* from *S. pruinosa*^22^, *Bacillus subtilis* from *Arthrochemum macrostachyum*^23^.

Up to our knowledge, all three isolates; *Providencia rettgeri* (P. rettgeri DW3), *Bacillus licheniformis* (B. licheniformis DW4) and *Salinicoccus sesuvii* (S. sesuvii DW5) were first time isolated from *T. nilotica*, *S. pruinosa* and *S. pruinosa* respectively, on the other hand, Providencia rettgeri, Bacillus licheniformis and Salinicoccus sesuvii were isolated from Suaeda fruticosa^24–26^. Plant-associated halotolerant bacterium Paenalcaligenes suwonensis (P. suwonensis DW7) were isolated for the first time from plants (*A. macrostachyum*), only isolated from patients^27^ and plastic wastes^28^. Bacillus amyloliquefaciens (B. amyloliquefaciens DW6) were isolated before recorded in^23^ .Providencia rettgeri from rhizospheric soil of tomato^29^, Bacillus licheniformis isolated from Cyamopsis tetragonoloba^30^, Salinicoccus hispanicus from Tamarix gallica^31^, Bacillus amyloliquefaciens from watermelon^32^ and Paenalcaligenes sp from soybean^33^.

Few studies have been published on faba beans, response to salinity by using the bacterial-mycorrhizal-legume tripartite symbiosis in saline conditions but no study has been published about the response of faba beans to salinity stress in the presence of PGPBs. The present study aims to isolate novel Plant-associated halotolerant bacteria, which were morphologically, biochemically, and molecularly identified. To investigate the potential of Plant-associated halotolerant bacteria in enhancing plant growth under salinity stress, with a specific focus on *Vicia faba* L. Mariout-2 as a model plant, and to assess the effectiveness of individual and/or co-inoculation with these bacteria as a novel bio-approach to mitigate the negative impact of soil salinity on plant growth.

## Results

### Isolation and purification of plant-associated halotolerant bacteria

**Figure 1:**
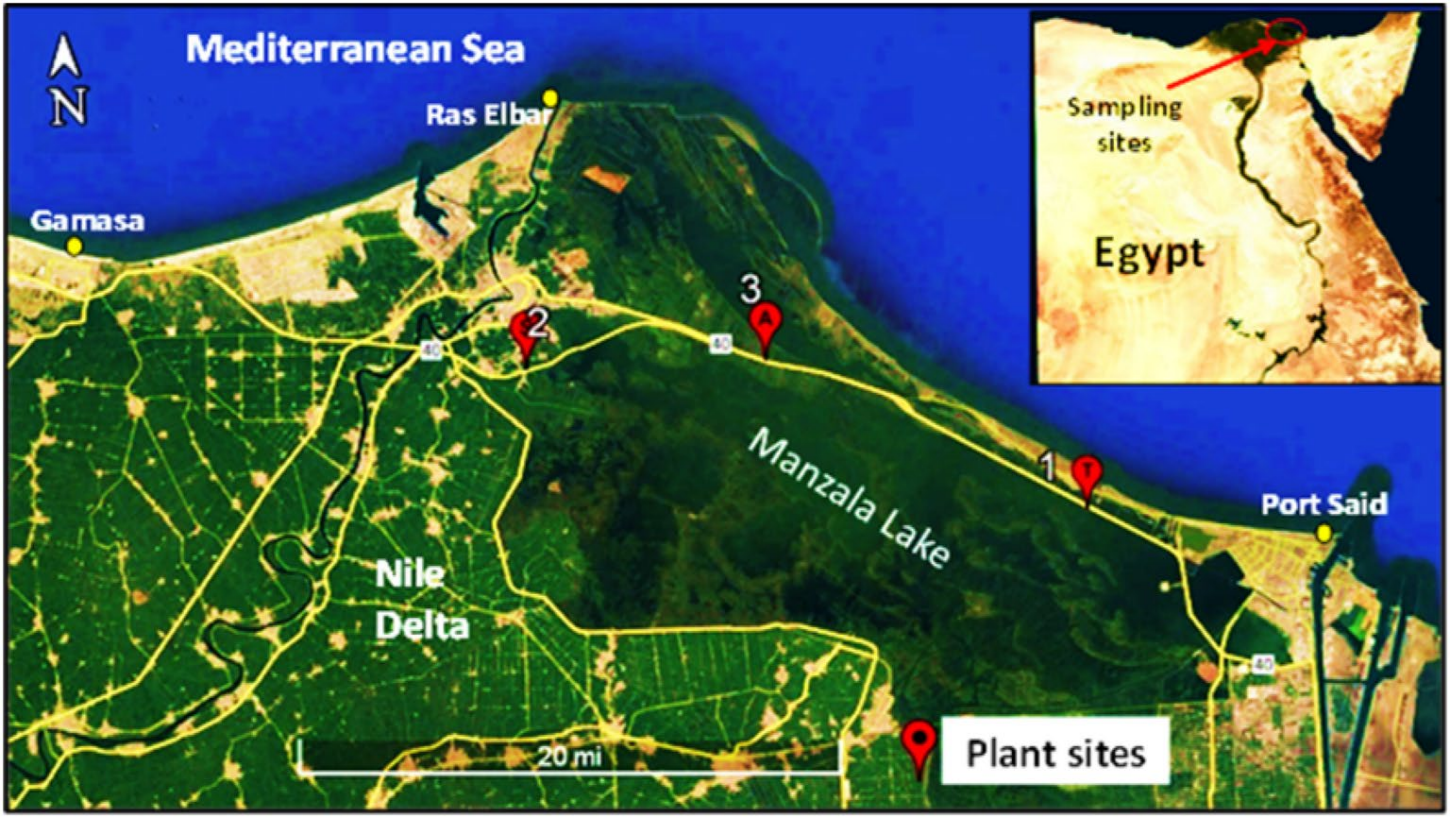
Map showing the site of samples collection, 1: *Tamarix nilotica*, 2: *Suaeda pruinosa* and 3: *Arthrocnemum macrostachyum*(Google Earth Pro version; 7.3, https://earth.google.com/web/@-0.21047219,-4.7998464,-5072.24236397a,19499383.57429804d,35y,0h,0t,0r/data=OgMKATA).

**Figure 2:**
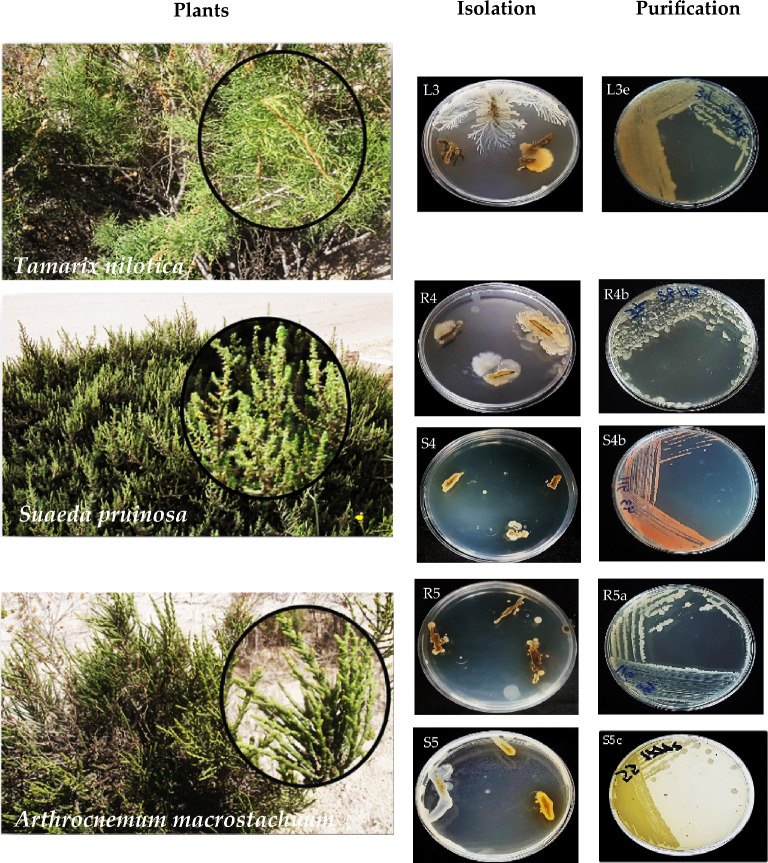
Isolation and purification of Plant-associated halotolerant bacteria from the root, stem, leaves, and rhizosphere of *Tamarix nilotica*,* Suaeda pruinosa* and *Arthrocnemum macrostachyum*. R: Root, S: Stem and L: Leaves. L3e: Leaves of *T. nilotica*, R4b and S4b: Roots and Stems of *S. pruinosa* and R5a and S5c: Roots and Stems of *A*. *macrostachyum.*

**Figure 3:**
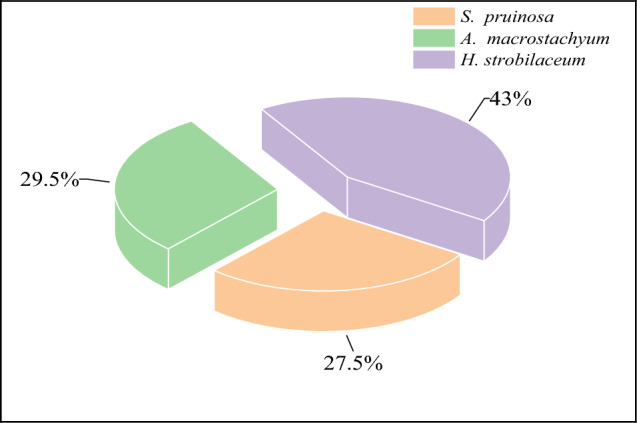
Distribution percentage of Plant-associated halotolerant bacteria in halophytes *Tamarix nilotica*, *Suaeda pruinosa,* and *Arthrocnemum macrostachyum.*

**Figure 4:**
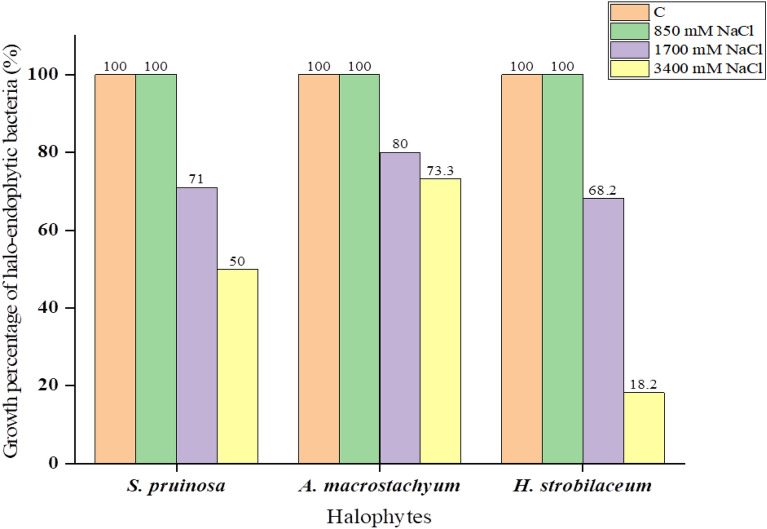
Growth percentage of plant-associated halotolerant bacteria from *Tamarix nilotica*, *Suaeda pruinosa*, and *Arthrocnemum macrostachyum* under different 850, 1700, and 3400 mM NaCl concentrations on LB agar media after 48 h at 28 °C.

Forty Plant-associated halotolerant bacteria were isolated and purified from plant parts of three halophytes *T. nilotica, S. pruinosa* and *A. macrostachyum* collected from the Mediterranean Deltaic coast, Dakahlia, and Damietta Governorates (Figs. 1 and 2), found maximum count of 15 (37.5%) in *A. macrostachyum* whereas minimum count of 11 (27.5%) obtained from *T. nilotica* and 14 (35%) from *S. pruinosa* (Figs. 3, 4 and Table 1).

**Table 1:** The count of bacteria isolated from the root, stem, leaves and rhizosphere of three halophytes, and the count of grown bacteria on different NaCl concentrations (mM).

Plant	Plant parts	Isolates count	Total count	NaCl concentration (mM)			
				170	850	1700	3400
*Tamarix nilotica*	Stem S3a, S3b, S3c, S3d and S3e	5	11	5	5	3	3
	Leaves L3a, L3b, L3c, L3d, L3e and L3f.	6		6	6	5	3
*Suaeda pruinosa*	Root R4a, R4b, R4c, R4d, R4e and R4f.	6	14	6	6	4	2
	Stem S_4_a and S_4_b	2		2	2	1	1
	Leaves L_4_a, L_4_b and L_4_c	3		3	3	2	1
	Rhizoshere Rh_4_a, Rh_4_b and Rh_4_c	3		3	3	3	3
*Arthrocnemum macrostachyum*	Root R_5_a, R_5_b and R_5_c	3	15	3	3	2	2
	Stem S_5_a, S_5_b, S_5_c and S_5_d	4		4	4	4	3
	Leaves L_5_a,L_5_b,L_5_c and L_5_d	4		4	4	2	2
	Rhizoshere Rh_5_a,Rh_5_b,Rh_5_c and Rh_5_d	4		4	4	4	4

### Screening of tolerant of plant-associated halotolerant bacteria

At 850 mM NaCl concentration, all 40 plant-associated halotolerant bacteria grew normally while 30 (75%) isolates grew afforded up to 1700 mM NaCl, and 24 (60%) isolates grew and tolerated up to 3400 mM NaCl concentration. Seven isolates L3e, R4b, S4b, Rh4b, Rh4c, R5a and S5c that tolerated up to 3400 mM NaCl concentration in media were selected for further investigations based on their fast growth. Overall, the growth rate of plant-associated bacteria decreased with increasing concentration of NaCl in the media (Table 1).

### Preliminary experiment of *Vicia faba* Mariout-2 seeds primed into plant-associated halotolerant bacterial isolates

The Increase in salinity concentration from 75, 150 up to 300 mM NaCl, the decrease in the percentage of germination, root length and shoot length *of Vicia faba* Mariout-2. The maximum percentage of germination was 100% as found in seeds treated with plant-associated halotolerant bacteria R5a and S5c, seeds treated with plant-associated halotolerant bacteria L3e, S4b, R5a showed a maximum root length of 8.4 ± 0.57, 9.4 ± 0.75 and 8.7 ± 0.67 cm respectively and seeds treated with plant-associated halotolerant bacterium R4b showed the maximum shoot length 25.4 ± 0.64 cm. The minimum percentage of germination of 71% was found in seeds treated with R5a, while seeds treated with C_2_ (50%) showed the minimum root length and shoot length of 0.9 ± 0.14 cm and 0.5 ± 0.00 respectively. Seeds treated with five plant-associated halotolerant bacteria L3e, R4b, S4b, R5a and S5c showed the maximum percentage of germination, root length and shoot length at 75, 150 and 300 mM NaCl compared to positive control (C_1_(freshwater)) and negative control (C_2_ (saline)). Of seven isolates, five Isolates were chosen for further study depending on their positive effects on *Vicia faba* L. mariout-2 beans growth under salinity stress (Table 2).

**Table 2:** Percentage of germination, root and shoot length of *Vicia faba* Mariout-2 treated with plant-associated bacteria under different salt concentrations.

Treatments			Percentage of germination	Root Length (cm ± SD)	Shoot Length (cm ± SD)
control	C_1_ (fresh)		100%	5.0 ± 0.53^ghi^	22.5 ± 2.5^ab^
	C_2_ (saline)	75 mM	100%	4.6 ± 0.53^ghi^	13.7 ± 1.9^fg^
		150 mM	100%	3.3 ± 0.36^i^	7.1 ± 1.87^ij^
		300 mM	100%	0.9 ± 0.37^j^	0.5 ± 0.00^l^
L3e		75 mM	100%	8.4 ± 1.51^abc^	22.6 ± 1.54^ab^
		150 mM	100%	5.1 ± 0.58^fghi^	17.4 ± 2.34^de^
		300 mM	85%	4.6 ± 0.69^ghi^	5 ± 0.40^ijk^
R4b		75 mM	100%	7.6 ± 1.96^abcd^	25.4 ± 1.69^a^
		150 mM	85%	5.3 ± 0.36^efghi^	15.6 ± 1.67^ef^
		300 mM	85%	4.9 ± 0.18^ghi^	8.1 ± 1.79^hi^
S4b		75 mM	100%	9.4 ± 1.97^a^	21.8 ± 1.95^bc^
		150 mM	100%	6.5 ± 1.06^cdefg^	17.5 ± 1.22^de^
		300 mM	85%	4.9 ± 0.18^ghi^	5.4 ± 0.67^ijk^
Rh4b		75 mM	85%	7.2 ± 1.31^bcdef^	23.9 ± 1.84^ab^
		150 mM	85%	5.4 ± 0.67^efgh^	10.5 ± 0.96^gh^
		300 mM	85%	4.8 ± 0.56^ghi^	3.9 ± 0.73^kl^
Rh4c		75 mM	100%	6.4 ± 0.67^cdefgh^	22.3 ± 2.23^abc^
		150 mM	100%	6.1 ± 0.95^defgh^	15.9 ± 2.14^def^
		300 mM	71%	4.4 ± 0.88^ghi^	4.1 ± 0.47^jk^
R5a		75 mM	100%	8.7 ± 1.77^ab^	21.4 ± 1.48^bc^
		150 mM	100%	7.9 ± 1.54^abcd^	12.9 ± 1.48^fg^
		300 mM	100%	4.9 ± 0.44^ghi^	7.4 ± 1.59^hij^
S5c		75 mM	100%	6.1 ± 0.89^defgh^	19.1 ± 1.43^cd^
		150 mM	100%	7.4 ± 1.48^abcde^	15.6 ± 0.67^ef^
		300 mM	100%	4.4 ± 0.37^hi^	5.9 ± 0.55^ijk^

Value: mean ± standard deviation (s.d.). Means shared the same letter(s) are not significantly different, Tuckey test (*p* ≤ 0.05).

### Morphological and molecular identification of plant-associated halotolerant bacteria

**Figure 5:**
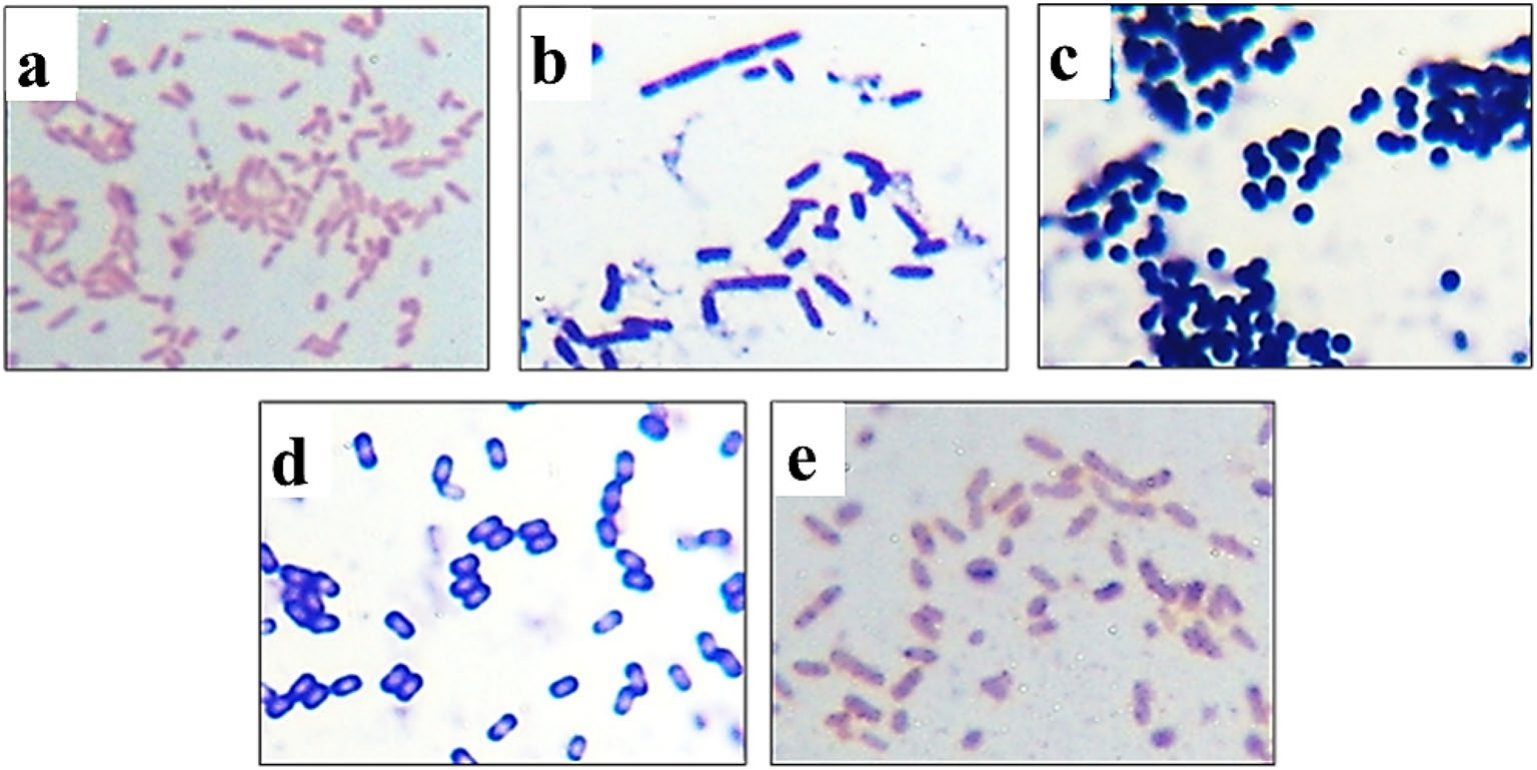
Microscopic images of five plant-associated bacterial isolates following Gram staining are coded as follows:: (**a**) L3e (**b**) R4b (**c**) S4b (**d**) R5a (**e**) S5c (1000X oil immersion lens).

Morphological characteristics of plant-associated halotolerant bacteria isolated from *T. nilotica, S. pruinosa* and *A. macrostachyum*; Shape, Size, Elevation, Margin, Surface/Texture, Opacity, Color, Odor and Gram stain summarized in Fig. 5 and Table 3. Four plant-associated halotolerant bacteria had bacilli-shaped structures and one bacteria was coccus-shaped.

**Table 3:** Morphological and cultural characteristics of plant-associated halotolerant bacteria isolated from *J. acutus, T. nilotica, S. pruinosa and A. macrostachyum*.

Plant	Isolates	Shape	Size	Elevation	Margin	Surface /Texture	Opacity	Color	Odor	Gram stain
*T. nilotica*	L3e	Circular	Medium	Convex	Entire	Smooth	Opaque	Brown	With odor	G-ve bacilli
*S. pruinosa*	R4b	Circular	Medium	Flat	Entire	Smooth/Viscid	Translucent	Creamy-white with EPS	With odor	G + ve bacilli
	S4b	Circular	Medium	Convex	Lobate	Smooth/Butyrous	Opaque	Orange	With odor	G + ve cocci
*A. macrostachyum*	R5a	Irregular	Large	Flat	Entire	Smooth	Opaque	Creamy white	With odor	G + ve bacilli
	S5c	Circular	Large	Flat	Entire	Wrinkle/Membranous	Opaque	Creamy white	With odor	G-ve bacilli

**Figure 6:**
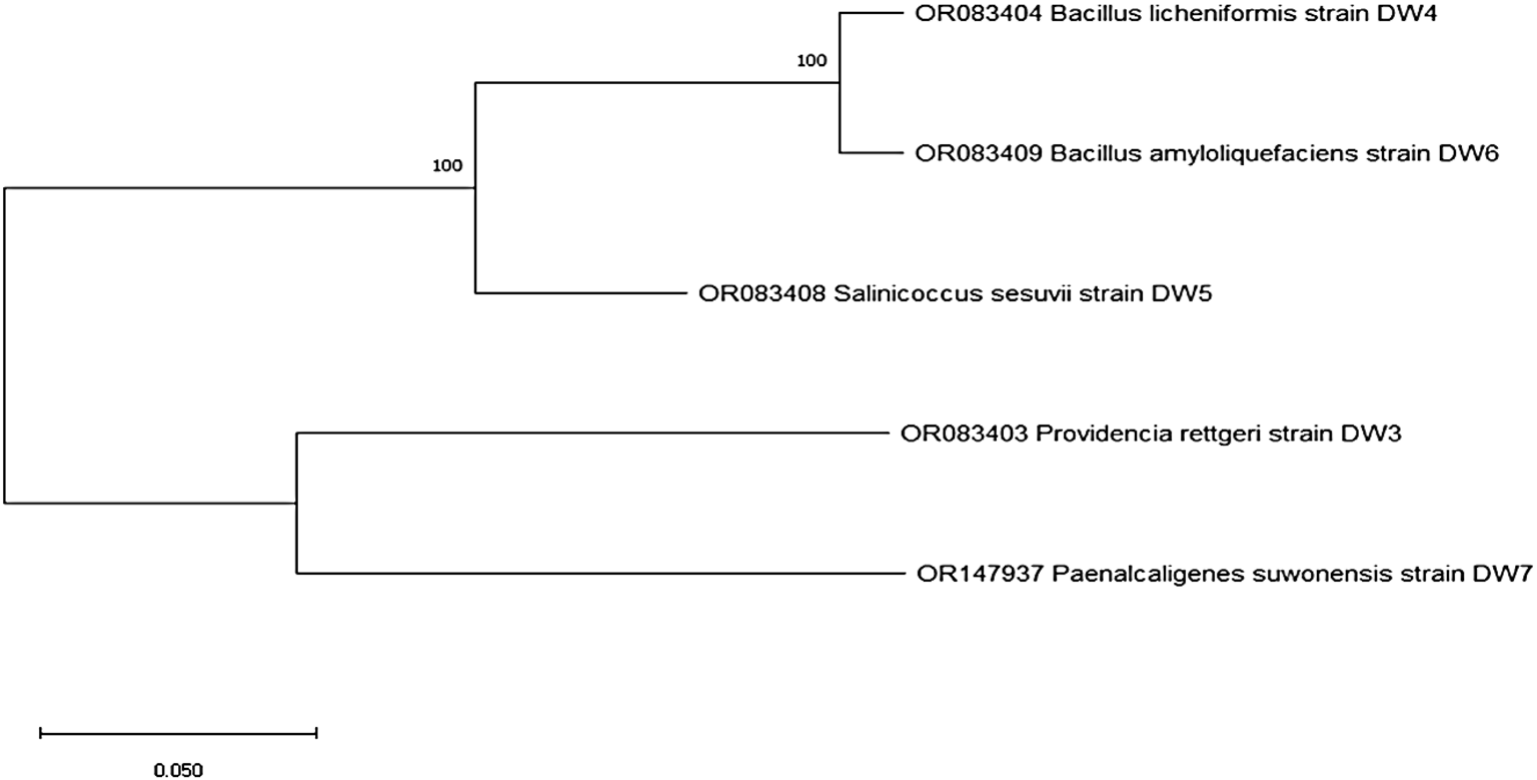
Phylogenetic dendrogram among five isolates conducted by MEGA11.

From sequencing analysis of the partial 16S rRNA region and BLAST analysis utilizing the NCBI database (Table 4), the isolate L3e (1386 bp) showed similarity (100%) with *Providencia rettgeri* so it has been given the name *Providencia rettgeri* DW3 and accession number of gene bank database OR083403, the accession number OR083404 belongs to the isolate R4b (1410 bp) showed identity (100%) to *Bacillus licheniformis* and this isolate has been given name *Bacillus licheniformis* DW4, the isolate S4b (1399 bp) showed high identity (100%) to *Salinicoccus sesuvii* so, it has been given name *Salinicoccus sesuvii* DW5 with accession number OR083408 on gene bank database, R5a (1399 bp) showed homology (100%) to *Bacillus amyloliquefaciens* so*,* it has been given name *Bacillus amyloliquefaciens* DW6 with accession number OR083409 on gene bank database and the isolate S5c (1371 bp) showed high identity (99.49%) to *Paenalcaligenes suwonensis* so, it has been given name *Paenalcaligenes suwonensis* DW7with accession number OR147937on gene bank database as illustrated in Table 4. The phylogenetic dendrogram illustrated the correlation among four isolates conducted by MEGA11 (Fig. 6).

**Table 4:** Molecular characterization of plant-associated halotolerant bacteria. isolated from *T. nilotica, S. pruinose* and *A. macrostachyum*.

Isolates ID	Plant-associated halotolerant Bacteria	NCBI Accession number	Homolog sequences	(Sequence identity %)	Phylum
L3e	*Providencia rettgeri* DW3	OR083403	*Providencia rettgeri*	100%	Enterobacteria
R4b	*Bacillus licheniformis* DW4	OR083404	*Bacillus licheniformis*	100%	Firmicutes
S4b	*Salinicoccus sesuvii* DW5	OR083408	*Salinicoccus sesuvii*	100%	Firmicutes
R5a	*Bacillus amyloliquefaciens* DW6	OR083409	*Bacillus amyloliquefaciens*	100%	Firmicutes
S5c	*Paenalcaligenes suwonensis* DW7	OR147937	*Paenalcaligenes suwonensis*	99.49%	b-Proteobacteria

As far as we know, all four isolates; *Providencia rettgeri* (*P. rettgeri* DW3)*, Bacillus licheniformis* (*B. licheniformis* DW4)*, Salinicoccus sesuvii* (*S*. *sesuvii* DW5) and *Paenalcaligenes suwonensis* (*P. suwonensis* DW7) were first time isolated from *T. nilotica, Suaeda pruinose, Suaeda pruinose* and *A. macrostachyum* respectively.

### In vitro assessments of plant growth promoting criteria of plant-associated halotolerant bacteria

#### Plant growth promoting criteria (PGP criteria)

**Figure 7:**
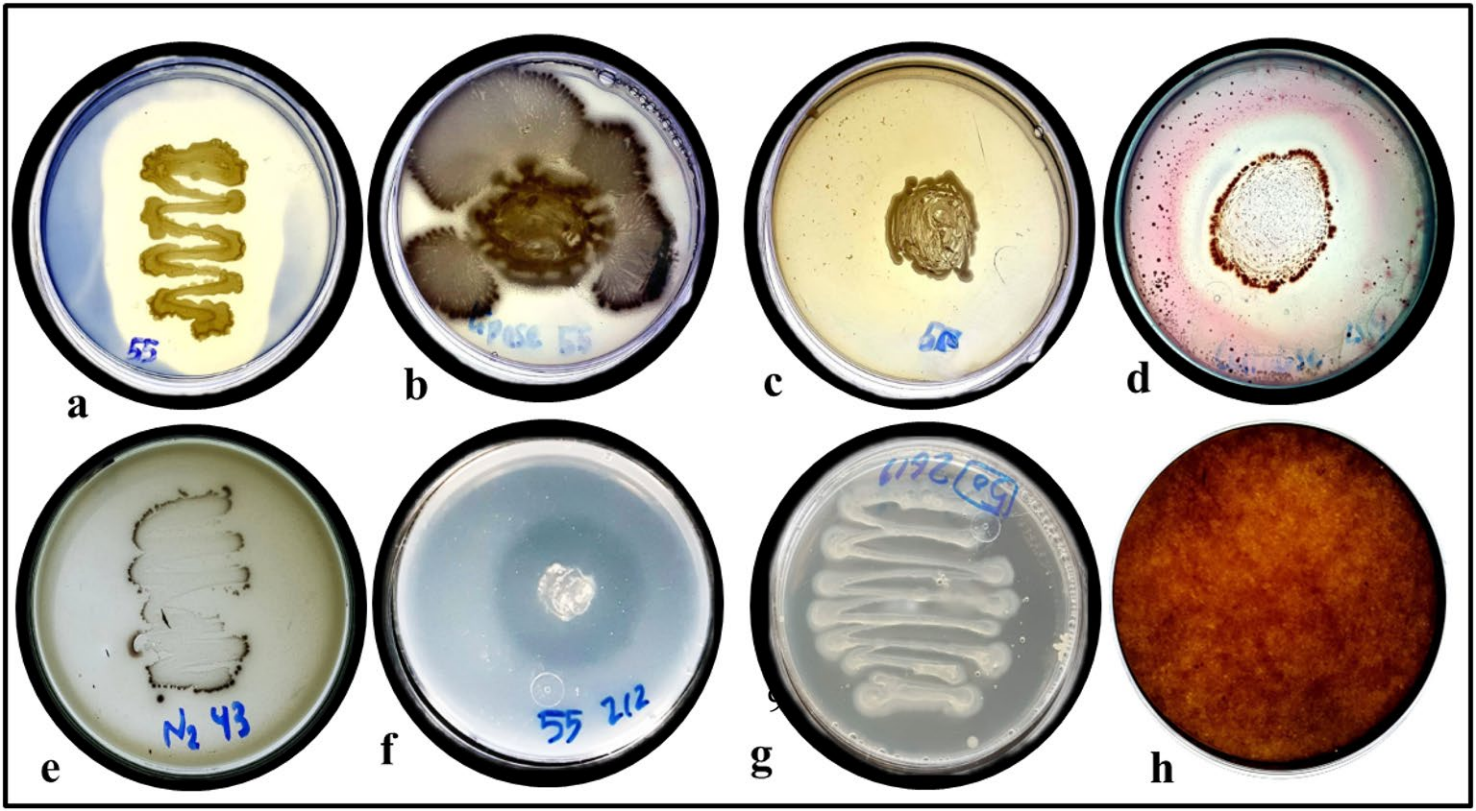
Plant growth promotion criteria and enzyme activity of plant-associated halotolerant bacteria isolated from S. pruinose and *A. macrostachyum* (**a**) amylse, (**b**) lipase, (**c**) protease, (**d**) cellulase, (**e**) preliminary experiment for nitrogen fixation, (**f**) phosphate solubilization, (**g**) exopolysaccharides and (**h**) HCN.

Indole acetic acid (IAA), Gibberellic acid (GA-3), exopolysaccharides (EPS), Hydrogen cyanide (HCN), ammonia production and siderophores production are quantitative assays, but preliminary experiment for nitrogen fixation and phosphate solubilization capacity are qualitative assays for studying the plant growth-promoting traits of 4 plant-associated halotolerant bacterial isolates. In the primary screening tests, all isolates showed nitrogen fixation and phosphate-solubilization capacity. Isolate *P. rettgeri* DW3 showed a maximum production of HCN 1484 μg ml^−1^, *B. licheniformis* DW4 showed a maximum production of EPS 25 mg ml^−1^ then *P. suwonensis* DW7 showed 16.667 mg ml^−1^, and isolate, *S. sesuvii* DW5 showed a maximum production of ammonia and siderophores 196.272 mg ml^−1^ and 76%, *B. amyloliquefaciens* DW6 showed a maximum production of, IAA 210 μg ml^−1^, GA-3 345.6 μg ml^−1^, respectively (Fig. 7 and Table 5).

**Table 5:** Plant growth promotion criteria and enzyme activity of plant-associated halotolerant bacteria isolated from *S. pruinose* and *A. macrostachyum.*

*DW3*	Tests	*P. rettgeri*	*B.licheniformis* DW4	*S. sesuvii* DW5	*B.amyloliquefaciens* DW6	*P. suwonensis* DW8
Quantative assays	IAA(μg/ml ± SD)	97 ± 1.5^e^	181 ± 1.01^b^	117 ± 1.5^d^	210 ± 1.07^a^	121 ± 1.1^c^
	GA3(μg/ml ± SD)	178 ± 0.96^d^	134 ± 0.55^e^	283 ± 2.26^b^	345 ± 1.05^a^	201 ± 0.51^c^
	EPS(mg/ml ± SD)	2.3 ± 0.30^d^	25 ± 1.00^a^	3.4 ± 0.30^c^	2.1 ± 0.30^d^	16.6 ± 0.45^b^
	HCN(μg/ml ± SD)	1484 ± 1.07^a^	133 ± 1.52^e^	164 ± 1.08^d^	223 ± 0.50^b^	215 ± 1.5^c^
	NH_4_ (mg/ml ± SD)	116 ± 1.00^c^	98 ± 1.52^e^	196 ± 1.01^a^	187 ± 2.00^b^	105 ± 1.5^d^
	Siderophores(% ± SD)	38 ± 0.025^d^	48 ± 0.035^c^	77 ± 0.026^a^	75 ± 0.030^b^	77 ± 0.032^a^
Qualitative assays	N_2-_fixation	+ ve	+ ve	+ ve	+ ve	+ ve
	PO_4_ Solubilization	+ ve	+ ve	+ ve	+ ve	+ ve
	*Enzymatic criteria*					
	Amylase	+ ve	+ + + ve	+ + ve	+ ve	+ + + ve
	Lipase	+ ve	+ + + ve	+ + ve	+ ve	+ + + ve
	Protease	+ ve	+ ve	+ + + ve	+ ve	+ + + ve
	Cellulase	+ + + ve	+ ve	+ + + ve	+ + + ve	+ + + ve

Value: mean ± standard deviation (s.d.). Means shared the same letter(s) are not significantly different, Tuckey test (*p* ≤ 0.05).

N_2-_fixation results based on preliminary evidence.

Isolate *B. amyloliquefaciens* DW6 showed a maximum production of all PGP criteria but a minimum in EPS production.

#### Enzymatic activity

Plant-associated halotolerant isolates' enzymatic activity demonstrated that amylase, lipase, protease, and cellulase activity was present in 100% of the isolates. Figure 7 and Table 5 displays the detailed results.

### Synergistic interaction among plant-associated halotolerant bacteria

The isolates' normal growth activity demonstrates that there is no possible antagonism between any of the examined pairs of bacteria. studied. Table 6. showed synergism between the five isolates that can grow normally together.

**Table 6:** Synergistic and antagonistic relation among plant-associated bacterial isolates.

Bacterial isolates	***P. rettgeri* DW3**	***B. licheniformis *DW4**	***S. sesuvii* DW5**	***B. amyloliquefaciens* DW6**	***P. suwonensis* DW7**
***P. rettgeri* DW3**	ND				
***B. licheniformis* DW4**		ND			
***S. sesuvii* DW5**			ND		
***B. amyloliquefaciens* DW6**				ND	
***P. suwonensis* DW7**					ND

### Seed priming

**Figure 8:**
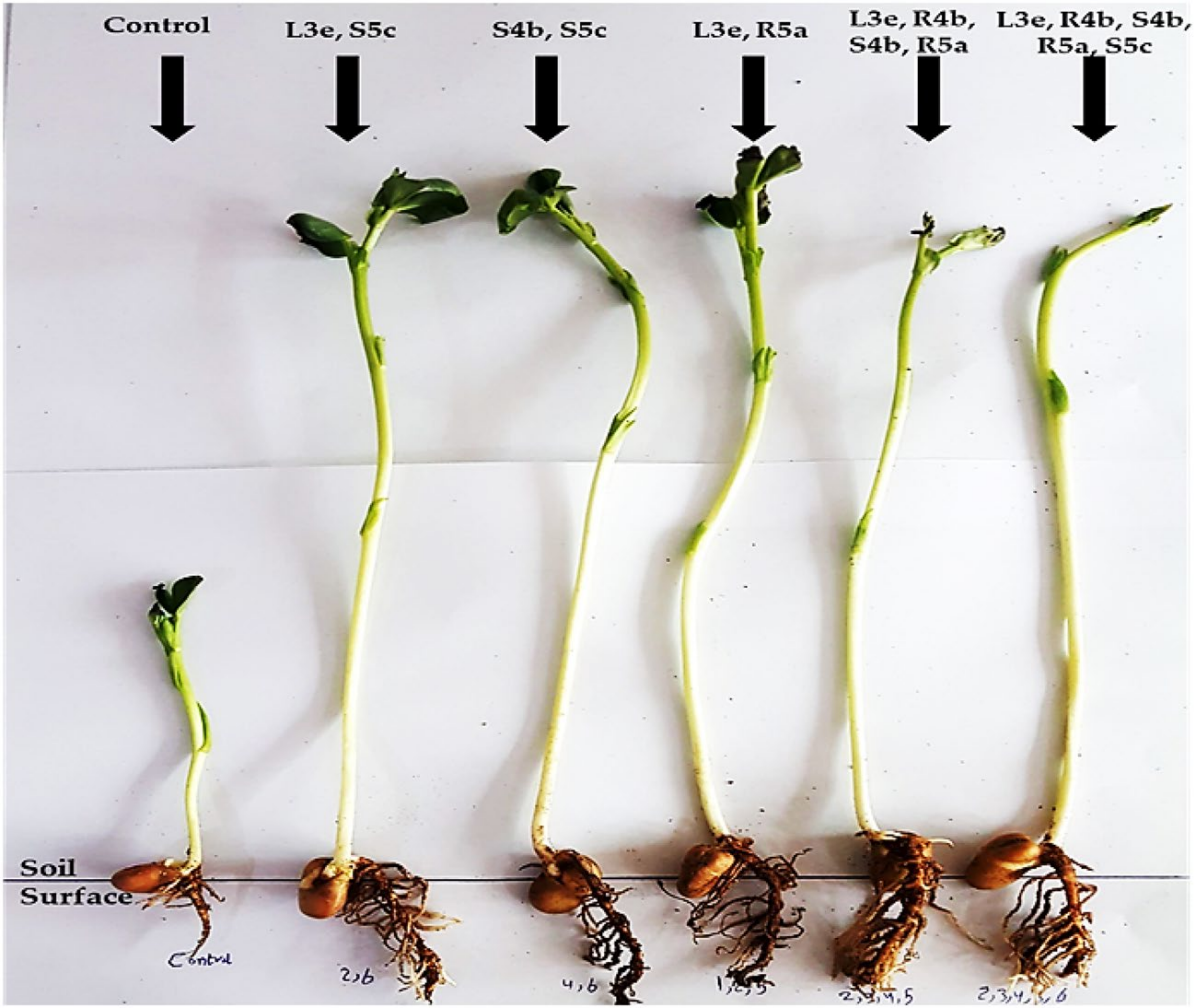
Germinating seed bioassay of *Vicia faba L. Mariout-2* seeds treated with different mixtures of bacterial isolates compared to the control after 2 weeks. L3e, S5c: *P. rettgeri* DW3*, P. suwonensis* DW7; S4b, S5c: *S. sesuvii* DW5, *P. suwonensis* DW7; L3e,R5a: *P. rettgeri* DW3*, B.amyloliquefaciens* DW6; L3e, R4b, S4b, R5a: *P. rettgeri* DW3*, B.licheniformis* DW4*, S. sesuvii* DW5*, B.amyloliquefaciens* DW6; L3e, R4b, S4b, R5a, S5c: *P. rettgeri* DW3*, B.licheniformis* DW4*, S. sesuvii* DW5*, B.amyloliquefaciens* DW6, *P. suwonensis* DW7.

The resulting synergistic plant-associated halotolerant bacteria were employed in seed biopriming and seed inoculation., five out of sixty-four showed the best growth under salinity conditions of 150 mM NaCl after 2 weeks compared to control which showed a lower rate in growth parameters and reported in Fig. 8 and Table 7.

**Table 7:** Root, shoot length and number of leaves of *Vicia faba* Mariout-2 treated with mixtures of plant-associated bacteria under 150 mM salt concentration.

Treatments	Root length mean ± SD (cm)	Shoot length mean ± SD (cm)	Number of leaves mean ± SD
Control	6 ± 0.8^b^	11.7 ± 1.52^b^	6 ± 1.00^d^
*P. rettgeri* DW3*, P. suwonensis* DW7	7.7 ± 0.47^ab^	22.3 ± 2.3^a^	14 ± 0.57^a^
*S. sesuvii* DW5, *P. suwonensis* DW7	7.5 ± 0.41^ab^	21.3 ± 2.3^a^	13 ± 0.57^bc^
*P. rettgeri* DW3*, B.a myloliquefaciens* DW6	8 ± 0.00^a^	21.7 ± 1.5^a^	12 ± 0.57^c^
*P. rettgeri* DW3*, B.licheniformis* DW4*, S. sesuvii* DW5*, B.amyloliquefaciens* DW6	7.3 ± 0.47^ab^	23 ± 0.00^a^	12 ± 0.00^c^
*P. rettgeri* DW3*, B.licheniformis* DW4*, S. sesuvii* DW5*, B.amyloliquefaciens* DW6, *P. suwonensis* DW7	8 ± 0.00^a^	22.7 ± 1.15^a^	14 ± 0.00^ab^

Value: mean ± standard deviation (s.d.). Means shared the same letter(s) are not significantly different, Tuckey test (*p* ≤ 0.05).

## Discussion

Studies on plant-associated bacteria have drawn more and more attention from researchers, because of their simpler isolation and identification, and also available molecular biology technologies. Plant-associated generate a variety of compounds with biological activity that are advantageous to medicines, industry, and agriculture^34^. plant-associated halotolerant bacteria have huge potential in agriculture to lessen the environmental effect of chemical fertilizers while also promoting the growth of plants by synthesizing plant hormones such as IAA, gibberellins, and cytokinins. Microorganisms and plants are capable of creating advantageous interactions in their natural environment and may build positive connections with each other^35^.

In this study, forty plant-associated bacterial strains were isolated from the halophytes; *T. nilotica*, *S. pruinosa* and *A. macrostachyum* roots, stems, leaves and rhizosphere as the main sources of plant-associated bacteria. Five isolates were molecularly identified by 16srRNA as *Providencia rettgeri* DW3 (OR083403.1), *Bacillus licheniformis* DW4 (OR083404.1), *Salinicoccus sesuvii* DW5 (OR083408.1), *Bacillus amyloliquefaciens* DW6 (OR083409.1) and *Paenalcaligenes suwonensis* DW7 (OR147937.1), all belonging to Firmicutes, which reported the most plant-associated phyla in addition to phyla enterobacteria and b-proteobacteria^36^. As far as we know, no one isolated the same three isolates; *Providencia rettgeri, Bacillus licheniformis* and *Salinicoccus sesuvii* from the same halophytes; *T. nilotica, S. pruinosa and S. pruinosa* respectively, while isolated from *Suaeda fruticosa*^24–26^. *Paenalcaligenes suwonensis* isolated from *A. macrostachyum,* was the first time to make* a* symbiotic relationship with plant, reported only two *Paenalcaligenes* sp isolated from patients^27^ and from plastic debris^28^.

According to the present investigation, all of the plant-associated bacterial isolates grew normally in LB medium with 170 mM (1%) sodium chloride. The plant-associated halotolerant isolates were grown at progressively increasing sodium chloride concentrations, and all isolates grew normally at 850 (5%) and 1700 mM (10%) NaCl, while increasing NaCl concentration up to 3400 mM (20%) NaCl, which led to reducing the bacterial growth, indicating that our isolates can tolerate salt stress better than other studies that screened the similar strains; *Providencia rettgeri* and *Bacillus* sp*.* isolated from *Suaeda fruticosa* can tolerate up to 10% and 9%, respectively^37,38^.

In order to determine how the bacterial isolates affected the seeds germination and growth, a number of different physiological characteristics were evaluated. The five isolates *P. rettgeri* DW3, *B. licheniformis* DW4, *S. sesuvii* DW5, *B. amyloliquefaciens* DW6 and *P. suwonensis* DW7 increase root and shoot length of *Vicia faba* under salinity stress at higher levels of seawater concentration compared to control. Other studies on related topics have validated these results on *Vicia faba*^23^ and Maize^26^.

All our five plant-associated halotolerant bacterial strains have a significant influence in enhancing seed germination and plant growth through GA3 and IAA production when L-tryptophan was present because plant roots generate tryptophan, which plant-associated halotolerant bacteria can absorb as a precursor for IAA formation. The microbial IAA combines the symbiotic link between plants and microbes, and it is thought to be the primary member of the auxins family. The bacterial strains were evaluated for the production of phytohormones such as GA3 and IAA^39^. It not only accelerates the growth of plants but also has a function in stimulating the germination process of seeds and tubers, speeds up the development of roots and xylem, improves lateral initiation, controls the rate of vegetative development and adventitious root formation, and promotes the synthesis of pigments and metabolites^39^. These findings were supported by multiple other investigations that demonstrated IAA synthesis by related strains obtained from various plants e.g., *Suaeda fruticosa*^37^, *Spergularia marina*^23,26^ and *Arachis hypogea*^25^. Gibberellins are growth regulators for plants that cause a number of developmental events in them, including germination, stem elongation, and senescence^40^. According to the results of this study, Isolate R5a generates more GA3 than earlier studies that tested similar strains isolated from *Capsicum annuum*^41^, *Oryza sativa*^42^ and Potato^43^.

The most important nutrient for *Vicia faba* is nitrogen, which is also a key component of chlorophyll, the substance that enables plants to use solar energy to produce sugars from water and carbon dioxide. It is also a significant portion of amino acids, the building blocks of proteins. In order to convert atmospheric nitrogen into a form that can be used, a specialized enzyme called nitrogenase is present in nitrogen-fixing bacteria^44^. Ammonia is regarded as a reliable source of nitrogen in addition to the nitrogen fixation process^45^. This study's findings on identical plant-associated bacterial strains obtained from various plants show that all strains have fixed nitrogen and created ammonia like in *Triticum aestivum*^46^, *Sasamorpha borealis*^47^, *Suaeda fruticosa*^37^ and rhizosphere of potato^48^.

According to Khalaf and Raizada^49^, phosphate is the second-most essential element for plant growth since it promotes the development of roots, flowering initiation, and the synthesis of protein. Plant-associated are one of the microorganisms that supply phosphate to plants^50^. Phosphate-solubilizing plant-associated bacteria may transform insoluble phosphates into soluble forms for plants through the processes of acidification, chelation, exchange reactions, and the creation of organic acids^51^. In this investigation, all five isolates can solubilize phosphate, which is verified by the findings of Nabti, et al.^48^, who investigated the capacity of *B. licheniformis* and Bacillus sp to solubilize phosphate and Bakelli, et al.^37^ also investigated the ability of *Providencia rettgeri* to make phosphate solubilization. However, the findings of Prashanth, et al.^52^ and Ye, et al.^36^ revealed that *B. licheniformis* MML2501 isolated from the rhizosphere of *Polygonum hydropiper* couldn’t solubilize phosphate.

EPS protects microorganisms against harsh circumstances including high temperatures, drought, and UV radiation, as well as storing nutrients and increasing tolerance to hazardous substances. All five isolates used in this investigation can produce EPS especially two isolates *Bacillus licheniformis* DW4 and Paenalcaligenes *suwonensis* DW7 generate more than previous research that tested the identical strains of soil-isolated bacteria found in other investigations^53^.

The release of hydrogen cyanide (HCN) by plant-associated bacteria is a bio-control action against pathogens that are regarded as indirect plant growth-promoting features (Singh, 2015). In this work, all isolates were positively producing HCN, especially *Providencia rettgeri* DW3 produced the highest amount of HCN, On the other hand, the results described by Amaresan, et al.^54^and ^41^ that none of the isolates showed the production of HCN.

Iron is an important nutrient resource. It acts as a cofactor in a number of enzymes that are needed for critical metabolic processes such as respiration, photosynthesis, and fixation of nitrogen^55^. Siderophore-producing rhizobacteria enhance plant health by boosting iron intake, inhibit the growth of other microorganisms by releasing their antibiotic molecule, and prevent the growth of pathogens by reducing the iron availability to the pathogen, typically fungi that are unable to consume the complex iron siderophore^56^. In this study, *Salinicoccus sesuvii* DW5, *Bacillus amyloliquefaciens* DW6 and *Paenalcaligenes suwonensis* DW7 were the highest among all isolates tested to produce siderophores units. In literature, Sharma, et al.^57^ and Dragojević, et al.^58^ characterized different types of siderophores produced by the same organism which supports the obtained data, others reported the absence of siderophores production by the same strain *Bacillus licheniformis* and *Providencia rettgeri*^37^.

According to Gao, et al.^59^, microorganisms' lytic enzymes used for polymer hydrolysis also act as extra bio-control agents and help in plant growth. In this study, all five isolates produce amylase, lipase, protease and cellulase enzymes, the findings that are validated by other investigations on related plant-associated bacterial strains isolated from various plants from the rhizosphere of potato^48^, the roots of soybean^57^, *Suaeda fruticosa*^37^ and *Capsicum annuum*^41^. On the other hand, the results described by Devi, et al.^41^ showed that *B. licheniformis* BECL5 isolated from *Capsicum annuum* couldn’t produce amylase, lipase, protease, and cellulase enzymes and *Bacillus* sp. couldn’t produce amylase and cellulase^38^.

Plant growth-promoting plant-associated halotolerant bacteria were used in this study to bio-prime seeds, and the combination of strains demonstrated an effective improvement in the percentage of seed germination. Plant growth-promoting strains also generate bioactive substances and hormonal stimulation, both of which contribute to plant promotion and disease defense^36^. The results of the co-culture study^60^ were in agreement with the Khan^61^ study of seed treatment and the investigation of seed priming carried out by Mahmood, et al.^62^. Bacterial communities and co-culture generate unique biologically active substances and secondary metabolites that boost plant health and productivity^63^. After co-inoculation, the synergistic impact boosts the effectiveness of other PGPB, leading to higher plant growth. In this study, the analysis was performed in a laboratory to test the effect of mixes of the five isolates (64 treatments on the growth of *Vicia faba* Mariout-2 leads to improving seed germination percentage, shoot and root length, all growth parameters were higher than control but the combined microbial treatments efficacy were better than the dual and individual treatments as it maximizes the IAA, GA-3 and all PGP criteria production in mixes more than single as supported by the work of Mahgoub, et al.^23^ and Khan, et al.^38^.

## Conclusions

In conclusion, this study successfully isolated and characterized a diverse group of plant-associated halotolerant bacteria using a comprehensive approach that included morphological, biochemical, and molecular analyses. The investigation revealed a range of novel bacteria exhibiting potential plant growth-promoting activities. Significantly, some of the isolated bacteria demonstrated the capacity to induce plant growth promotion and enhance salinity tolerance, marking a promising breakthrough in the field of agricultural research. This finding underscores the potential of these plant-associated halotolerant bacteria as valuable resources for improving crop growth under salinity stress, a critical concern in agriculture. The outcomes of this research pave the way for further exploration and application of these beneficial bacteria in agriculture, with the potential to contribute to sustainable and resilient farming practices in saline environments. Additionally, the study highlights the importance of harnessing microbial diversity for addressing contemporary agricultural challenges and emphasizes the need for future investigations to delve deeper into the mechanisms behind these bacteria's plant growth-promoting abilities under salinity conditions. In summary, this study not only expands our understanding of plant-associated halotolerant bacteria but also offers a promising avenue for the development of innovative agricultural strategies aimed at mitigating the detrimental effects of soil salinity on plant growth and crop productivity.

## Materials and methods

### Isolation and purification of plant-associated halotolerant bacteria from plants and rhizosphere

In January 2022 (18°/5 °C), *T. nilotica* (Ehrenb) Bunge. (31°17′23.32″ N, 32°10′12.29″ E), *S. pruinosa* Lange (31°22′42.30″ N, 31°49′3.44″ E) and *A. macrostachyum* (Moric.) K.koch. (31°22′42.96″ N, 31°58′0.43″ E) were collected from the Mediterranean Deltaic coast, Dakahlia, and Damietta Governorates. All halophytes were grown naturally as natural resources and didn’t require a permit for collection. *Tamarix nilotica* is classified as least concerned by IUCN, while *S. pruinosa* and *A. macrostachyum* are not evaluated yet. Experimental research and field studies on plants (either cultivated or wild), including the collection of plant material, were performed in accordance with relevant institutional, national, and international guidelines and legislation and comply with the IUCN Policy Statement on Research Involving Species at Risk of Extinction and the Convention on the Trade in Endangered Species of Wild Fauna and Flora, available from: https://portals.iucn.org/library/efiles/documents/PP-003-En.pdf*.* The plant samples and soil were placed in clean plastic bags and then brought to the bacteriology lab for microbiological experimentation.

The plant-associated bacteria were isolated from fresh samples of root, stem, and leaf after being cleaned in running tap water for 2 min before being washed in 70% ethanol for 2 min. The samples were then rinsed for 1 min in 2% sodium hypochlorite for surface sterilization. The leaves were then given a final 2 min washing in sterile distilled water before drying^64^. After pretreatment, the samples were chopped into smaller pieces with sterile scissors and placed in contact with the surface of the Luria–Bertani (LB) agar plates with 170 and 340 mM NaCl concentrations. The water from the previous rinse was streaked over the LB medium and incubated at 28 °C for 24 h to determine the efficiency of surface sterilization. For isolation from the rhizosphere, the root-attached soil was collected in sterilized distilled water to make a soil solution, after soil particle precipitation, the supernatant was used as an inoculum onto LB agar plates with 170 and 340 mM NaCl concentration. Following incubation, colonies of bacteria with unique morphologies were selected and streaked on LB. agar plates, and incubated for 48 h at 28 °C. On LB agar slants, each of the selected isolates was sub-cultured and maintained at 4 °C.

### Halotolerance test

Luria–Bertani (LB) agar medium supplemented with 850, 1700, and 3400 mM NaCl was inoculated by the forty isolates of plant-associated halotolerant bacteria for 48 h at 28 °C. Bacterial growth was monitored every 24 h and documented.

### Seed priming preliminary experiment

Based on results obtained from the halotolerance test, seed priming was conducted to investigate the effect of seven different bacterial plant-associated isolates, L3e, S5c, S4b, Rh4b, Rh4c, R5a, and R4b on germination of *Vicia faba* Mariout-2 grown under normal and salinity conditions. Healthy *Vicia faba* mariout-2 seeds were received from the Field Crop Research Institute (FCRI), Agricultural Research Centre, Giza, Egypt. The used equipment and seedlings were adequately sterilized before commencing the germination test. Homogeneous seeds were surface sterilized by first cleaning them under a running tap. After sterilizing the seeds for 2 min with 70% ethyl alcohol, they were washed three times with distilled water. The seeds were also sterilized for 5 min in a 3% sodium hypochlorite solution before being cleaned three times in sterile distilled water ^18^. Surface sterilized *V. faba* seeds were soaked for 24 h into 50 ml of 0.8 optical density bacterial suspensions at 600 nm as well as soaked in distilled water as control. The bacterial-soaked seeds were planted after 24 h of incubation in 84-well Peat Moss trays (one seed/each) at a room temperature of 25–30 °C with seven replica, positive and negative control seeds were soaked in LB medium and watered by Nile water and seawater systems. The watering system with different ratios of seawater and Nile water 1: 7, 1: 3 and 1: 1 (75, 150 and 300 mM) was applied to seeds primed with bacterial isolates. Positive control seeds were watered with Nile water, while the negative control was irrigated with different salt concentrations, one tray for each seawater concentration. The trays were left at natural day/night period at room temperature for 7 days. Germination percentage, and root and shoot length were measured.

### Characterization of plant-associated halotolerant bacteria

#### Morphological characterization (cell shape)

The cell morphological properties of the pure cultures were examined microscopically after staining by Gram stain ^65^.

#### Molecular identification of plant-associated halotolerant bacteria

The five isolates were molecularly identified by 16S rRNA gene sequencing. Solgent purification beads were used to extract bacterial DNA from the pure culture. The mixture was mixed using a vortex (Globe Scientific-500) and incubated at 95 °C for 15 min in a heat block. The universal primers 27F 5'-AGA GTT TGA TCC TGG CTC AG-3' and 1492R 5'-GGT TAC CTT GTT ACG ACT T-3' were used to amplify the 16S rRNA gene of the five isolates ^66^. The amplifications of PCR were carried out using the Solgent PCR conditions listed below: firstly, 30 cycles at 95 °C for 20 s, then the temperature was reduced to 50 °C for 40 s, then 72 °C for 1 min 30 s, and the final extension was at 72 °C for 5 min. PCR products were analyzed using 1% agarose gel electrophoresis and purified by a purification kit from Solgent and then analyzed on an ABI 3730XL DNA Analyzer. Using Finch (version 1.4.0), the collected sequence data were edited and aligned, and a contiguous consensus sequence was created. The National Centre for Biotechnology Information (NCBI)'s Basic Local Alignment Search Tool (BLAST) software (http://blast.ncbi.nlm.nih.gov) algorithm was used to search for homology using an aligned contiguous consensus sequence of the 16S rRNA gene. The isolates' 16S rRNA gene sequences were phylogenetically studied using the neighbor-joining method with NCBI run blast ^67^. Data collected from sequencing were entered into the NCBI Gene Bank database to receive accession numbers.

#### PGP criteria

##### IAA detection

According to Loper and Schroth^68^, IAA production was assessed by inoculating a plant-associated bacterial isolate on LB broth medium supplemented with (1.02 g L^−1^) 1-tryptophane and incubated in an orbital shaking incubator at 150 rpm for 3 days at 28 °C. One mL of the culture's supernatant was added to 2 mL of Salkowski reagent (60% perchloric acid, 3 mL 0.5 M FeCl3 solution) after the culture had been centrifuged at 7000 rpm for 3 min. IAA quantification can be determined by pink color absorption at 530 nm. IAA concentration was evaluated using a standard curve of 3-Indol acetic acid obtained from Sigma Aldrich between 10 and 100 g ml^−1^^69^.

##### GA-3 detection

GA-3 production was measured after inoculating a bacterial isolate in LB broth medium and incubating it in a shaker at 150 rpm for 14 days at 28 °C. Following a 5000 rpm centrifugation, 15 mL of the supernatant was added, along with 2 mL of zinc acetate reagent, followed by 2 mL of 10.6% potassium ferrocyanide solution and centrifuged at low speed for 2 min. Five mL of supernatant was mixed with 5 mL of 30% HCl and incubated at 25 °C for 75 min. The absorbance was determined at 254 nm, and a blank of 5 mL of 5% HCl was utilized. The concentration of gibberellin was estimated in several concentration ranges (100–1000 μg mL^−1^) using gibberellic acid as a standard for creating a standard curve as described with slight modifications^70^ using gibberellic acid as a benchmark.

##### Exopolysaccharide production

Using nutrient agar medium supplemented with high sucrose content (5%), plant-associated halotolerant bacterial isolates were examined qualitatively for their capability to produce exopolysaccharides (EPS). The isolates were streaked on the plates and left to incubate for 4–7 days at 28 °C. Visually, over the streak, a thick, viscous substance developed. The isolates demonstrated that this viscous substance was indictable for polysaccharide synthesis^71^. 100 mL of EPS production medium was prepared and inoculated by plant-associated halotolerant bacterial isolates and incubated in an orbital shaking incubator at 150 rpm at 28 °C for 7 days^18^. After incubation, the broth culture was centrifuged at 4000 rpm for 30 min at 4 °C to separate bacterial cells. For precipitation of EPS, the supernatants were treated with three times the amount of cold acetone and left at 4 °C overnight. The extraction of EPS was carried out by centrifugation at 4000 rpm for 10 min, at last, the pellets were weighed to assess EPS concentration^72^.

##### Hydrogen cyanide production

Plant-associated bacteria were inoculated into King's B medium supplemented with 4.4 g L^−1^glycine and incubated for 48 h at 28 °C^73^. As HCN was synthesized, the indicator paper's color changed from deep yellow to reddish-brown^74^. The filter papers were then cut into small pieces and soaked in 2 mL of distilled water. The result was measured spectrophotometrically at 510 nm. The following equation was used to determine the concentration of HCN in (ppm).

$$\text{Total cyanides contents }(\text{ppm}) =396 \times \text{ A}510\text{ nm}$$

##### *Ammonia* production

For ammonia production, 10 mL of water peptone broth media were inoculated by plant-associated halotolerant bacterial isolates and then incubated for 4 days at 28 °C. Nessler's reagent (1 mL) was added to each tube to measure the ammonia accumulation. Color Development varied from a faint yellow, which indicates a minimum production of ammonia, to a brown color, which indicates a maximal production of ammonia. At 450 nm, the result was measured spectrophotometrically. The standard curve was created using ammonium sulfate to determine the ammonia content in each sample (0.6–15 mg L^−1^)^75^.

##### Preliminary test for nitrogen fixation

The streak plate technique was used to analyze the growth of the bacterial isolates in Jensen's agar medium (Sucrose, 20 g/L K_2_HPO_4_, 1.0 g/L MgSO_4_, 0.5 g/L NaCl, 0.5 g/L Na_2_MoO_4_, 0.005 g/L FeSo_4_, 0.1 g/L CaCo_3_, 2.0 g/L bacteriological agar–agar, 20 g/L) (Nitrogen-free media) for 4–7 days at 28 °C^76^.

##### Phosphate solubilization

The qualitative estimation of tricalcium phosphate solubilization by plant-associated halotolerant bacterial isolates was carried out on the Pikovskaya agar medium. The isolates were streaked onto Pikovskaya agar medium and after 5 days of incubation at 28 °C, their phosphate-solubilizing activity was determined. The formation of a clear zone surrounding the colony suggested the presence of inorganic phosphate solubilization^77^.

##### Siderophores production

The bacterial isolates were inoculated into MM9 broth media (15 mL). The cultures were incubated at 28 °C for 2 days. After 10 min of centrifugation at 5.000 rpm, 0.5 mL of the supernatant was combined with 0.5 ml of CAS (chrome azurol S) reagent, and the result was spectrophotometrically measured at 630 nm. The amount of siderophores was determined using the following equation by^78^.

$$\text{Siderophores units }\left(\text{\%}\right)=\frac{\text{Ar}-\text{As}}{\text{Ar}}\text{ x }100$$

where Ar = Absorbance of blank at 630 nm (CAS reagent), As = Absorbance of the sample at 630 nm.

#### Enzymatic activity

##### Amylase

To determine the amylase activity, plant-associated halotolerant bacteria were inoculated onto starch agar medium and incubated for 2–3 days at 28 °C, followed by flooding the plates with iodine solution. The plates were examined to have clear zones surrounding the colonies, which was interpreted as a positive response^65^.

##### Lipase test

Tween agar medium was used for the detection of lipase activity by inoculating the strain on the plates and incubating them for 2–4 days at 28 °C^79^. The idea of precipitation of calcium salt underpins this approach. Fatty acids are created by tween hydrolysis and mixed with the medium's calcium to form insoluble crystals close to the inoculation site. Lipase activity was indicated by white precipitation at the colony's perimeter^80^.

##### Protease test

Protease activity was measured on skim milk agar plates infected with the strain and incubated for 24 h at 28 °C. When a definite proteolytic zone was discovered around the colonies, it was established that the screened bacteria were able to produce protease^81^.

##### Cellulase test

Determination of cellulose activity was carried out by streaking on cellulose agar medium and incubated at 28 °C for 2–5 days^82^. After that, the plates were flooded with 0.2% aqueous Congo red and destained with 1M Sodium chloride for 15 min to examine the activity, as indicated by^83^.

### Co-culturing test

To study the synergism and the antagonism among *P. rettgeri* DW3, *B. licheniformis* DW4, *S. sesuvii* DW5, *B. amyloliquefaciens* DW6 and *P. suwonensis* DW7, LB solid medium plates were prepared and subsequently inoculated with bacterial isolates. The bacterial strains were cultivated on the same plate, with cross-growth between each pair of plant-associated bacteria to see if there was any evident antagonism between them. The plates were then incubated at 28 °C for 48 h.

### Second seed priming

The present experiment was carried out at the Bacteriology laboratory, Botany Department, Faculty of Science, Mansoura University, Egypt in 2023. The protocol was created in order to alleviate salt stress and improve the growth of *Vicia faba* Mariout-2 seeds under salinity conditions using plant growth-promoting bacteria (PGPB) application through seed priming in a mixture of bacterial suspensions.

Whole broad bean (*Vicia faba* Mariout-2) seeds were sterilized using the preceding methods. Under aseptic conditions, surface sterilization was performed on the seeds of *Vicia faba* Mariout-2. They were then immersed overnight in 50 ml of five bacterial suspensions (OD_600_ = 0.8) in single and different mixtures of 64 treatments. Seeds were soaked in distilled H_2_O for 24 h as a control*.* The soaked seeds were planted after 24 h of incubation, in trays with 84 wells filled with soil mixture (Peat Moss). Each well was planted with one treated seed at a temperature of 25–30 °C, each treatment with three replicas. The experiment was designed by irrigation with a seawater concentration of 150 mM NaCl and applied on the plant, one tray for all mixtures of isolates and three trays for the three replicas of *Vicia faba* Mariout-2 seeds. The trays were left at natural day/night conditions at a temperature range of 26–29 °C for 2 weeks and the number of leaves, and root and shoot length were then calculated.

### Statistical analysis

The obtained data were arranged in a randomized block design. After performing a one-way ANOVA, mean averages were compared based on the Tuckey test at probability ≤ 0.05. The software: CoStat (version 6.450, CoHort Software, Birmingham, UK) was used.

### Experimental research and field studies

On plants (either cultivated or wild), including the collection of plant material, were performed in accordance with relevant institutional, national, and international guidelines and legislation and comply with the IUCN Policy Statement on Research Involving Species at Risk of Extinction and the Convention on the Trade in Endangered Species of Wild Fauna and Flora, available from: https://portals.iucn.org/library/efiles/documents/PP-003-En.pdf.

## Data Availability

The data supporting this work are included in this published article.
